# Differences in signal contrast and camouflage among different colour variations of a stomatopod crustacean, *Neogonodactylus oerstedii*

**DOI:** 10.1038/s41598-020-57990-z

**Published:** 2020-01-27

**Authors:** Amanda M. Franklin, Justin Marshall, Adina D. Feinstein, Michael J. Bok, Anya D. Byrd, Sara M. Lewis

**Affiliations:** 1 Biology Department, Tufts University, Medford, MA 02155 USA; 2 School of Biosciences, The University of Melbourne, Parkville, Victoria 3010 Australia; 3 Sensory Neurobiology Group, Queensland Brain Institute, University of Queensland, Brisbane, Queensland 4072 Australia; 4 School of Biological Sciences, University of Bristol, Life Sciences Building, Bristol, BS8 1TQ UK; 5 Department of Biological Sciences, University of Maryland Baltimore County, Baltimore, MD 21250 USA

**Keywords:** Behavioural ecology, Evolutionary ecology

## Abstract

Animal colouration is often a trade-off between background matching for camouflage from predators, and conspicuousness for communication with con- or heterospecifics. Stomatopods are marine crustaceans known to use colour signals during courtship and contests, while their overall body colouration may provide camouflage. However, we have little understanding of how stomatopods perceive these signals in their environment or whether overall body coloration does provide camouflage from predators. *Neogonodactylus oerstedii* assess meral spot colour during contests, and meral spot colour varies depending on local habitat. By calculating quantum catch for *N. oerstedii*’s 12 photoreceptors associated with chromatic vision, we found that variation in meral spot total reflectance does not function to increase signal contrast in the local habitat. *Neogonodactylus oerstedii* also show between-habitat variation in dorsal body colouration. We used visual models to predict a trichromatic fish predator’s perception of these colour variations. Our results suggest that sandy and green stomatopods are camouflaged from a typical fish predator in rubble fields and seagrass beds, respectively. To our knowledge, this is the first study to investigate signal contrast and camouflage in a stomatopod. These results provide new insight into the function and evolution of colouration in a species with a complex visual system.

## Introduction

Animal body colouration is subject to opposing selective pressures. Predation often selects for patterns and colours that blend in with the background whereas communication selects for colours that have high contrast with the background^1,2^. Many strategies have evolved to deal with these opposing pressures. For instance, animals may use signal partitioning whereby body regions that are visible to predators provide camouflage and body regions hidden from potential predators are used for signalling. Male agamid lizards are cryptically coloured dorsally for camouflage from birds, but have conspicuous colours on their throat and chest that are visible to conspecifics during mate choice^3^. Other animals may exploit limitations to spatial resolution by using patterns that cannot be resolved at large viewing distances but are conspicuous at short viewing distances^4^. For example, Cinnabar caterpillars are brightly coloured and conspicuous at shorter viewing distances, but at larger viewing distances these colours blend together and match the background^5^. Alternatively, animals may communicate with conspecifics via signals that the predator cannot detect. For example, the Ambon damselfish, *Pomacentrus amboinensis*, uses UV face markings for species recognition^6^. Because several reef predators are UV-blind, including the larger fish species, this is considered a secret communication channel. Also, some stomatopods reflect circularly polarized patterns that may be involved in aggressive or mating displays^7,8^. To date, stomatopods are the only animal known to distinguish circular polarization cues. Many animals also alter their signalling behaviours when predators are present or most active. Guppies, *Poecilia reticulata*, perform visually conspicuous courtship behaviours during times of the day when predators are least active^9^. Lastly, some species can change colour depending on the context. A popular example are chameleons, which can change colour either to match their background^10^ or to become conspicuous during contests and courtship^11^. Research into the trade-off between camouflage and conspicuousness provides insight into the evolution of animal colouration and mechanisms to ameliorate the cost of conspicuous signals.

Investigations into signalling and camouflage should consider the visual system of the intended receiver, background colouration, and the lighting environment. Visual systems differ significantly in the number of spectral classes, some organisms having only a single photoreceptor type, whereas others, such as some butterflies and stomatopods, have between 10 to 20 photoreceptor classes^12,13^. Employing visual models that are based on receiver visual physiology allows predictions to be ecologically relevant. The signalling environment, including the background and lighting conditions, can also affect the perception of colours. For example, certain colours and patterns that appear highly conspicuous outside of natural conditions can match natural backgrounds through disruptive camouflage^14^. Furthermore, lighting conditions, such as filtering through a forest canopy or depth in water, can influence how colours are perceived^15,16^. Thus, studies which incorporate these factors into predictions of conspicuousness or camouflage are more likely to produce ecologically relevant predictions. However, it can be difficult to make predictions for animals with complex or unconventional visual systems.

**Figure 1:**
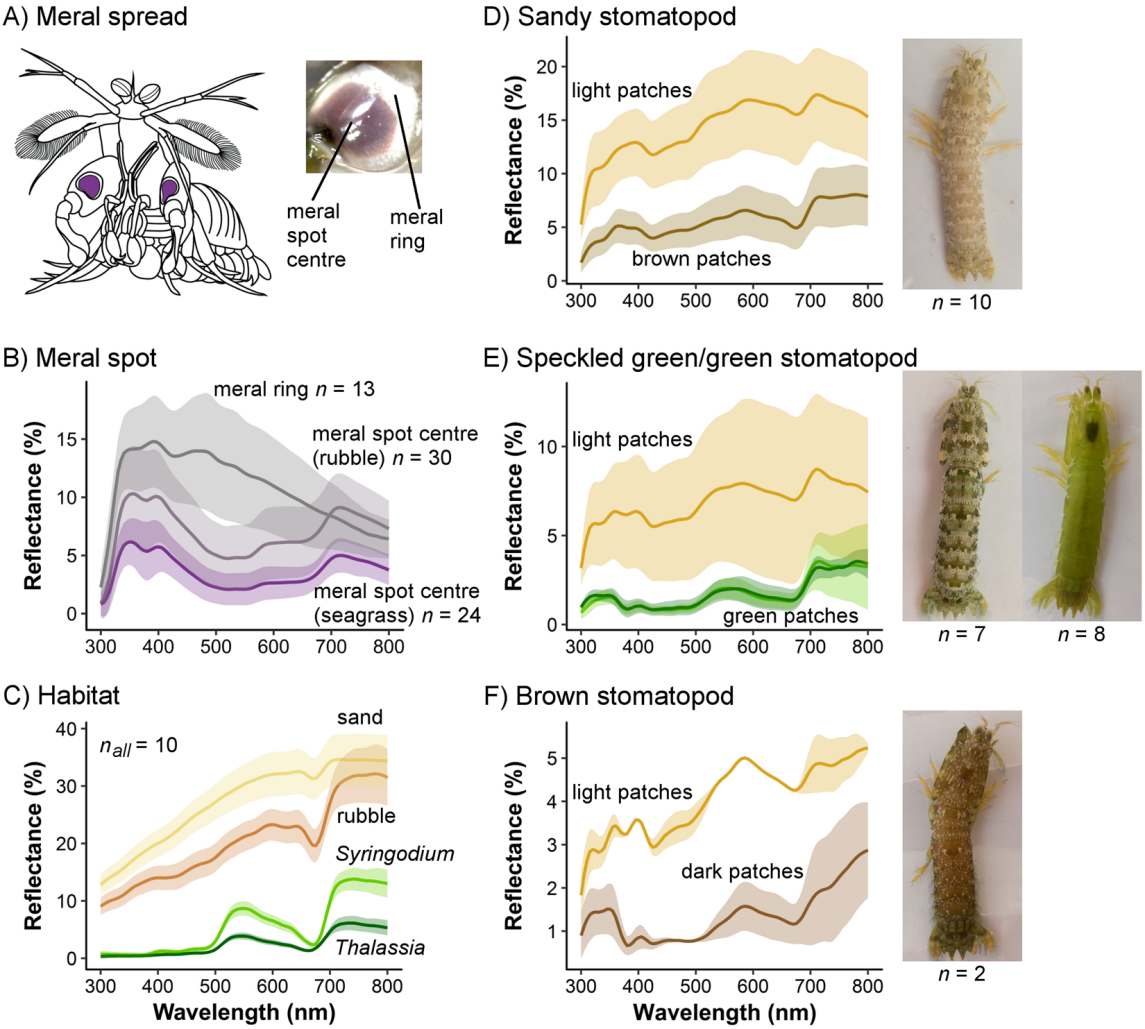
Spectra of stomatopod colours and background colours. (**A**) Schematic of a stomatopod performing the meral spread and displaying the meral spots (Adapted from A. M. Franklin et al., 2017, Behav. Ecol. 1329–1336. By permission of Oxford University Press on behalf of the International Society for Behavioral Ecology). Inset depicts the meral spot centre and meral ring. (**B**–**F**) Average spectra (±SD) of *Neogonodactylus oerstedii* meral spot centres and meral rings (**B**), background habitat (**C**) and *N. oerstedii* sandy (**D**), green and speckled green (**E**), and brown (**F**) dorsal colour variations. Images show representative examples of colour variant.

Stomatopods are marine crustaceans that have an unconventional visual system^13^. Their eyes have up to 20 photoreceptor classes, allowing them to see UV, visible and polarised light^7,13,17–19^. At least one species, *Odontodactylus scyllarus*, has circular polarisation vision, and there is morphological evidence for circular polarisation vision in other stomatopod species^7^. Unlike most other organisms, they do not appear to process visual information using opponent mechanisms, resulting in coarse colour discrimination^18^. Nonetheless, stomatopods have been shown to use colour signals in both courtship and agonistic encounters^20–26^. For example, during territorial contests, stomatopods increase agonistic behaviours in response to increased total reflectance of the meral spot^24^, a coloured patch exposed during the meral spread threat display (Fig. 1A). Furthermore, darker meral spots correlate with increased strike impulse, suggesting that meral spot colour indicates weapon performance^27^. For these colour signals to be effective in natural conditions, they should contrast with the background habitat, and with any adjacent colours on the stomatopod.

*Neogonodactylus oerstedii*^28^ are shallow water (<10 m), Caribbean stomatopods that exhibit habitat-specific variation in the colour of the meral spot^25^. Stomatopods collected from sandy, rubble habitats have lighter meral spots than those collected from adjacent seagrass habitats^25^. This variation in total reflectance (*c.f*. luminance) may increase contrast between the signal and the background to a stomatopod receiver. Here, we tested this prediction by modelling contrast between the meral spot and seagrass or rubble backgrounds, as perceived by a stomatopod receiver. We also examined whether contrast within the meral spot (*i.e*., between the coloured meral spot centre and the surrounding pale ring) may help conspecific receivers to assess meral spot centre total reflectance.

Some species of stomatopod also exhibit intraspecific variation in dorsal body colouration that encompasses colour, total reflectance, and pattern. In some cases, colouration is sexually dimorphic and, thus, likely to play a role in sexual selection or sex recognition^29,30^. In other instances, body colouration varies even within each sex. For example, *N. oerstedii* can be solid green, speckled green, speckled brown or speckled sandy coloured (Fig. 1D–F). This variation in pattern and colour likely functions to match the background substrate. In fact, many species of stomatopod, including Gonodactylids like *N. oerstedii*, can change colour depending on their local background^22,31,32^. However, this change is not instantaneous, but occurs over multiple moults. Although this variation in colour and pattern may reflect local background-matching, the camouflage function of stomatopod body colouration has yet to be investigated. *Neogonodactylus oerstedii* are likely preyed upon by reef fish such as triggerfish, snapper and wrasse^33^. Predation events can occur when stomatopods leave their refuge to search for food or mates^34^. Here, we assess *N. oerstedii* camouflage in terms of chromatic aspects (hue and saturation), total reflectance and pattern. To assess camouflage of different *N. oerstedii* colour variations in both seagrass and rubble habitats, we model perception of stomatopod colouration and background colouration by a trichromatic reef fish. We also investigate whether the meral spot is visible to a fish predator, or whether stomatopods may be using a secret communication channel (*i.e*., the meral spot is conspicuous to conspecifics, but not to a fish predator).

## Methods

### Study species and sample collection

*Neogonodactylus oerstedii*^28^ are Gonodactylid stomatopods found throughout the Caribbean in shallow waters (<10 m depth). They have broad spectral sensitivity, from the UV to the far red, and may have linear and circular polarisation vision. These stomatopods reside in cavities in coral rubble, rocks and conch shells (hereafter called ‘rubble’), where they process food, mate, brood eggs and avoid predators^34^. They will emerge from these refuges to forage for food and males will abandon their refuge to locate a mate^34^.

This research was conducted at the Smithsonian Institution’s research facility at Carrie Bow Cay, Belize (16°48′9′′N, −88°4′55′′W) in July 2015. We collected *N. oerstedii* from rubble found in shallow (<3 m deep) mixed seagrass beds (*Thalassia testudinum* and *Syringodium filiforme*) and adjacent rubble fields. Stomatopods were extracted from their refuge in rubble using a pick and then housed in 19 L white, plastic buckets with running seawater. Upon transfer to the lab, all stomatopods had their body length (tip of rostrum to tip of telson), wet weight and sex recorded. Each stomatopod was classified by body colouration as either brown, speckled green, solid green or sandy (Fig. 1D–F). Immediately before photographs and spectral measurements were to be recorded, stomatopods were euthanized by cooling in the freezer (−13 °C) for 30 minutes. This does not affect colour if stomatopods are kept wet. We also collected ten blades each of *T. testudinum* and *S. filiforme* seagrass, ten pieces of rubble, and ten samples of sand.

### Photographs

Stomatopods were photographed in the field in both seagrass and rubble habitats with their dorsal side visible (Fig. S1). Photos were taken of euthanised stomatopods between 11am to 2 pm on sunny days. We used a Canon G16 digital camera (Tokyo, Japan) set to record RAW files. White balance was set using an 18% grey card, and a waterproof colour card (DKG Color Tools, Boston, USA) was included in each photo. Focal length was kept constant at 6 mm (fully zoomed out). Stomatopods were weighed down using fishing sinkers and positioned in the appropriate habitat with their dorsal surface upwards.

### Spectral measurements

Colour measurements were recorded using a JAZ spectrophotometer (Ocean Optics, Dunedin USA) with a PX-2 pulsed xenon light source. Reflectance was recorded between 300 nm and 800 nm, and measured relative to a WS-1 white standard. The light source was positioned at 45° to the sample and the collector probe perpendicular to the sample to mimic natural conditions^35^. Both probes were 600 µm UV-VIS fibre optic cables (Ocean Optics, Dunedin USA) with collimating lenses attached to the end and were fixed in position to standardise distance between the probes and the sample. Measurements were recorded in a dark box so only the light source was illuminating the sample.

Multiple spectral measurements were recorded from every sample. For each habitat type (*S. filiforme, T. testudinum*, rubble and sand) we recorded three to five spectra. Spectra were recorded from the base of seagrass blades and the top surface of rubble pieces. For each stomatopod, we recorded seven spectral measurements of each colour present (one from the carapace, four from different abdominal segments, and two from the raptorial appendages). Therefore, stomatopods with speckled colouration (brown, sandy and speckled green) had 14 spectral measurements recorded, seven from light patches and seven from dark patches.

Spectra of meral spot centres (Fig. 1A) were recorded following the same methods, at the same field site, in 2014^25^. We recorded meral spot centre measurements from 24 stomatopods collected in seagrass habitat and 30 stomatopods collected from rubble beds. Two measurements were recorded from each meral spot. Meral ring measurements were recorded similarly, however, measurements were recorded at University of Maryland Baltimore County with a USB-2000 spectrophotometer and 400 µm UV-VIS fibre optic cables (Ocean Optics, Dunedin USA). These stomatopods (*n* = 13) were ordered commercially from KB Marine Life in Florida, USA. This is a different population of *N. oerstedii* to those at Carrie Bow Cay. We do not know whether meral ring spectral measurements are similar between Floridian and Belizean populations, or whether there are habitat differences in meral ring spectral reflectance. Therefore, comparisons involving the meral ring provide an indication of contrast but require further investigation to determine if these are biologically meaningful comparisons.

All spectra were smoothed with a loess smoother to remove noise. For all background samples, meral spot measurements and green stomatopods, we obtained one spectra for each sample by averaging all spectra within a sample. For, sandy, brown and speckled green stomatopods, we calculated two averages per sample, a light patch average and a dark patch average.

### Analysis

Analyses were conduction in R 3.5.3^36^.

#### Stomatopod signalling: meral spot

**Figure 2:**
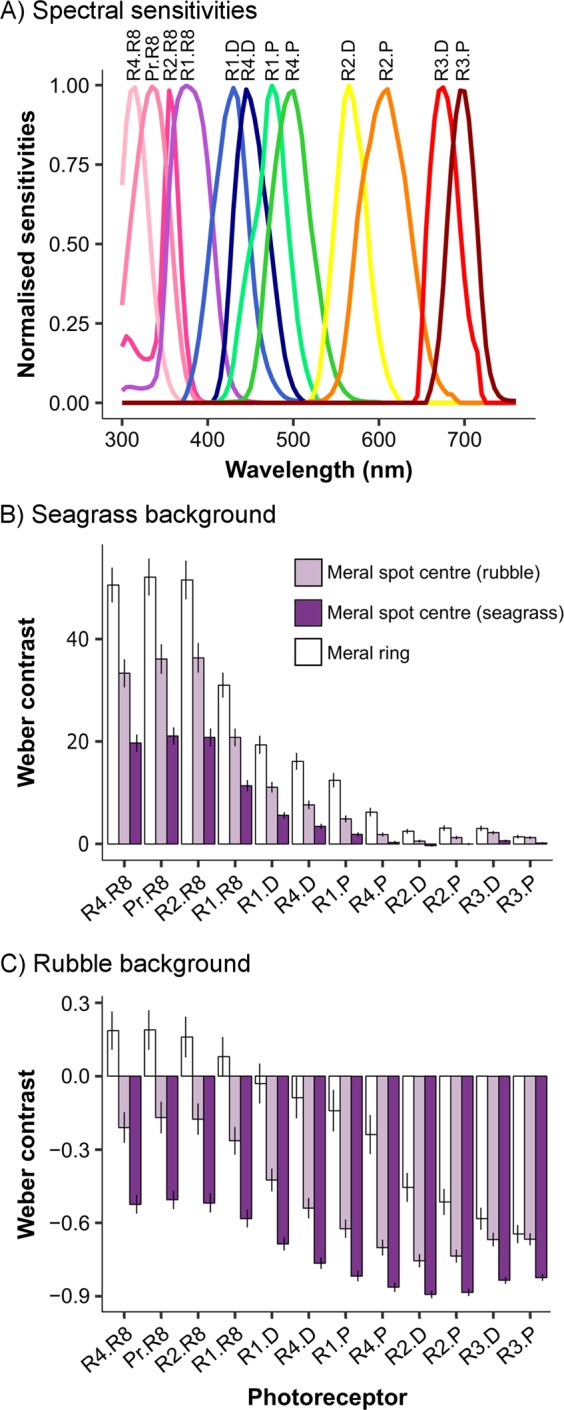
Stomatopod signalling: meral spot. (**A**) Spectral sensitivities of photoreceptors associated with chromatic vision in the stomatopod *Neogonodactylus oerstedii*. Adapted from Marshall *et al*.^17^ and Bok *et al*.^37^. (**B**–**C**) Weber contrast values for *N. oerstedii* meral spots (see Fig. 1B) when viewed horizontally against a seagrass (**B**) or rubble (**C**) background, as perceived by a conspecific. Contrasts were conducted for meral spot centres of stomatopods collected from rubble habitats (light purple) and seagrass habitats (dark purple). Photoreceptors are numbered by their position in the stomatopod eye’s midband row (R1-R4) or periphery (Pr) and whether they are dorsal (**D**), proximal (**P**) or R8 retinular cells.

We calculated Weber contrasts to investigate whether the variation in meral spot total reflectance between different habitats functions to increase signal contrast against the background to a stomatopod receiver. We did not compare receptor outputs because stomatopods do not appear to process chromatic information using a colour-opponent coding system^18^. Meral spot centres and meral rings were compared with either a rubble background or a *T. testudinum* background, to model the horizontal view of a stomatopod receiver. We elected to use rubble and *T. testudinum* because *N. oerstedii* reside in rubble pieces and *T. testudinum* is the most abundant seagrass species where we collected stomatopods. For each meral spot centre spectra, ring spectra, and habitat spectra, we estimated quantum catch for *N. oerstedii*’*s* 12 photoreceptors associated with chromatic vision (Fig. 2A). Spectral sensitivity curves were adapted from Marshall *et al*.^17^ and Bok *et al*.^37^. Quantum catch is an estimate of the number of photons each photoreceptor detects^38^ and can be calculated as:

1 $${q}_{i}={\int }_{300}^{800}{R}_{i}(\lambda )S(\lambda )I(\lambda )d\lambda $$

where *R*_*i*_(*λ*) is the spectral sensitivity of photoreceptor *i*, *S*(*λ*) is the reflectance of the sample and *I*(*λ*) is the irradiance. Integration was done over the sensitivity range of the stomatopod. Stomatopods that exhibited variation in meral spot colour with habitat were collected at <2 m depth^25^ so we calculated quantum catch using irradiance for 1 m depth on a tropical reef (Fig. S2)^39^.

For every photoreceptor, we then calculated Weber contrasts (*ω*_*i*_) for each stimulus (s) against each background (b) as:

2 $${\omega }_{i}=\frac{{q}_{i}^{s}-{q}_{i}^{b}}{{q}_{i}^{b}}$$

The stimulus was either (1) the meral spot centre from seagrass collected stomatopods, (2) the meral spot centre from rubble collected stomatopods or (3) the meral ring. The background was either (1) seagrass or (2) rubble. For each meral spot centre (rubble collected: *n* = 30; seagrass collected: *n* = 24) and meral ring (*n* = 13), we averaged these Weber contrasts to obtain one contrast value for each photoreceptor in each background type.

We also calculated Weber contrasts to investigate whether the meral ring functions to increase contrast within the meral spot, to a stomatopod receiver. In this case, the stimulus was either (1) the meral spot centre from seagrass collected stomatopods or (2) the meral spot centre from rubble collected stomatopods, and the background was the meral ring. Again, we averaged these Weber contrasts to obtain one contrast value for each photoreceptor for each sample.

#### Stomatopod camouflage: hue and saturation

To determine if stomatopods match the background hue and saturation, we modelled chromatic contrast from the perspective of a trichromatic fish predator. Modelled colours are plotted on chromaticity diagrams which depict variation in hue and saturation, independent of achromatic components (*i.e*., total reflectance). We used the spectral sensitivities of the Picasso triggerfish, *Rhinecanthus aculeatus* (Fig. S3), adapted from Pignatelli *et al*.^40^. This fish has a visual system representative of common coral reef fish that are likely predators of stomatopods^41^. First, we calculated quantum catch for the short wavelength sensitive (SWS; *λ* = 420 nm), medium wavelength sensitive (MWS; *λ* = 480 nm) and long wavelength sensitive (LWS; *λ* = 520 nm) photoreceptors. We modelled irradiance for a range of depths that *N. oerstedii* is commonly located: 0.5 m, 1 m, 3 m, 7 m and 10 m (Fig. S2). Irradiance measurements were adapted from Marshall *et al*.^39^. Integration was done over the range of sensitivity for the Picasso triggerfish, 340–700 nm.

Quantum catch values were transformed to a Von Kries adapted value by dividing quantum catch values by adapting background light:

3 $${v}_{i}=\frac{{q}_{i}}{{\int }_{340}^{700}{R}_{i}(\lambda )B(\lambda )I(\lambda )d\lambda }$$

where *B*(*λ*) is the background reflectance. We report results for both seagrass and rubble adapting background light. These results were then normalised by dividing by total photoreceptor quantum catch:

4 $${n}_{i}=\frac{{v}_{i}}{{\sum }_{i}{v}_{i}}$$

These corrections normalise photoreceptor response relevant to background radiance.

We calculated *x* and *y* coordinates to project the response onto a 2-dimensional chromaticity diagram for trichromats, called Maxwell’s triangle. The coordinates are calculated by:

5 $$x=\frac{{n}_{L}-{n}_{S}}{\sqrt{2}}$$

6 $$y=\sqrt{\frac{2}{3}}\frac{{n}_{M}-({n}_{L}+{n}_{S})}{2}$$

where *n*_*L*_ is the normalised quantum catch for the LWS photoreceptor, *n*_*M*_ is for the MWS photoreceptor and *n*_*S*_ is the SWS photoreceptor. This calculation assumes opponent mechanisms of colour processing. Distance from the centre of the triangle corresponds to saturation and other deviations related to hue.

We then estimated whether stomatopod colours contrasted with the background when viewed by a trichromatic fish predator using the receptor noise limited vision model^42^. This model calculates chromatic discrimination thresholds based on receptor noise. Following previous studies, we assumed a noise threshold of 0.05, and a receptor ratio of 1:2:2 (S:M:L)^43^. For this calculation, we compared all stomatopod colour samples with all background samples for each background type (*T. testudinum, S. filiforme*, rubble and sand). These quantum catch values and estimates of noise were substituted into Eq. 4 from Vorobyev and Osorio (1998)^42^ to estimate chromatic contrast, or just noticeable difference (JND) (implemented in R using the *pavo* package^44^). To investigate whether stomatopod colours differed from background samples we followed the two-step procedure detailed in Maia and White (2018)^45^. This procedure first tests whether samples are statistically different and then tests whether samples are perceptually different. To investigate statistical differences between samples, we used the chromatic contrast values calculated above to create distance matrices between stomatopod colour samples and background samples. We then tested the assumption of homogeneity of variances using a multivariate extension of Levene’s test for homogeneity^46^ implemented in R using ‘betadisper’ (package: vegan^47^). This was followed with PERMANOVAs to determine if samples are statistically separate (‘adonis’, package: vegan). To determine if samples are perceptually different to a fish predator, we used a bootstrap procedure to generate geometric means and 95% confidence intervals. Samples are considered distinct and likely to be discriminable if the results from the PERMANOVA are statistically significant and if the bootstrapped confidence intervals do not include the threshold for discrimination^45^. The threshold at which two colours are indistinguishable has not been tested in fish, but studies in other organisms consider values below 1 JND to be indistinguishable^48,49^. Thus, we considered stomatopods to camouflage from predatory fish (*i.e*., to ‘match’ the background) if PERMANOVA results were not significant, or if bootstrapped confidence intervals crossed one and mean JND values were less than three.

#### Stomatopod camouflage: pattern and total reflectance

The photographs of stomatopods taken in the field were used to assess whether a stomatopod’s pattern and total reflectance matches its background when perceived by a fish predator. We focussed on green (*n* = 11) and sandy (*n* = 9) stomatopods because these are the most common dorsal colourations in seagrass and rubble habitats, respectively. These were analysed using the micaToolbox plugin^50^ in ImageJ (v1.5)^51^. First, regions of interest were selected. In rubble habitat, the regions of interest were the stomatopod, and similarly sized areas of rubble pieces and sand (four of each habitat type). In seagrass habitat, the regions of interest were the stomatopod and similarly size areas of sand, clean *Thalassia* blades and *Thalassia* blades covered in silt (three of each habitat type). Fish likely use their double cones for luminance vision and pattern discrimination^52^. These cones usually are sensitive around 500 nm which corresponds to the green part of the spectrum^53^. Thus, patterns were analysed using the green colour channel of the image^54,55^ (Fig. S1). For each region of interest, we calculated total pattern energy, peak pattern frequency and mean luminance. Total pattern energy provides an estimate of pattern contrast, peak pattern frequency indicates primary pattern size and luminance indicates total reflectance or intensity^56,57^. Thus, we will refer to these variables as pattern contrast, pattern size and total reflectance.

We ran Linear Mixed Models (LMMs) to assess the difference in response variables (pattern contrast, pattern size and total reflectance) between stomatopods and background types (rubble habitat: sand and rubble pieces; seagrass habitat: sand, clean *Thalassia* blade and silty *Thalassia* blade). Each habitat (rubble or seagrass) was analysed separately with ‘item type’ (*i.e*. green stomatopod, sandy stomatopod, or background type) as a fixed effect. All response variables were log-transformed to meet normality assumptions and each model included *photo number* as a random effect to account for any differences between photos. Model fit was assessed using residual plots. Output from these models were used to calculate Cohen’s *d* for repeated measures designs^58,59^. This was calculated for contrasts between stomatopods (green or sandy) and background types (sand, rubble pieces, clean *Thalassia* blade and silty *Thalassia* blade). Cohen’s *d* provides an indication of the magnitude of difference between groups and we considered values of *d* < 0.8 to indicate a ‘match’ between the stomatopod and the background type^56,58^.

The datasets analysed during the current study are available from the corresponding author on reasonable request.

## Results

### Stomatopod signalling: meral spot

We examined whether habitat variation in meral spot colour (Fig. 1B) increases contrast with the respective habitat, as perceived by a stomatopod receiver. In seagrass habitats, meral spots appear lighter than the seagrass background (positive contrast values), whereas in rubble habitats meral spots appear darker than the rubble background (negative contrast values; Fig. 2). In the seagrass habitat (Fig. 2B), the meral spots of stomatopods collected from rubble habitats provide greater contrast than the meral spots of stomatopods collected from seagrass habitats (Fig. 2B). However, in rubble habitats, the meral spots of stomatopods collected from seagrass habitats contrast more with the background (Fig. 2C). This pattern applies across all 12 of *N. oerstedii*’s photoreceptors that are associated with chromatic vision.

Generally, the meral ring was brighter than the seagrass background and darker than the rubble background. However, three photoreceptors in the UV had greater quantum catch from the meral ring than from the rubble background (Fig. 2C). In seagrass habitats, the meral ring contrasted more with the background than meral spots of stomatopods collected from either habitat. In the rubble habitat, the meral ring tended to contrast less with the background than meral spots of stomatopods from either habitat.

**Figure 3:**
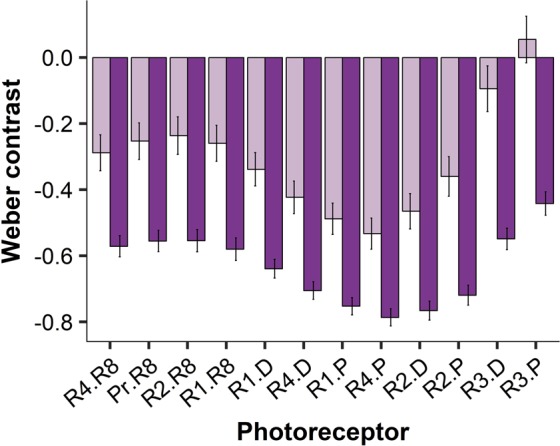
Stomatopod signalling: meral spot. Weber contrast values for *Neogonodactylus oerstedii* meral spot centres when viewed against the meral ring (see Fig. 1B). Meral spot centres for stomatopods collected from rubble habitats are light purple and meral spot centres for stomatopods collected from seagrass habitats are dark purple. Photoreceptors are numbered by their position in the stomatopod eye’s midband row (R1-R4) or periphery (Pr) and whether they are dorsal (D), proximal (P) or R8 retinular cells.

Meral rings may also provide contrast within the meral spot, assisting receiver stomatopods to assess meral spot centre colour. The darker meral spots from stomatopods collected in seagrass habitats contrast more with the meral ring than the lighter meral spots of stomatopods collected from rubble habitats (Fig. 3).

### Stomatopod camouflage: hue and saturation

We collected 27 *N. oerstedii* and classified them into four dorsal colour variations: sandy (*n* = 10), solid green (*n* = 8), speckled green (*n* = 7) or brown (*n* = 2). Spectral reflectance measurements of the light patches on patterned stomatopods (sandy, speckled green and brown) are similar in shape across all colour variations (Fig. 1D–F). The green patches on both green colour patterns are also extremely similar in shape and total reflectance (Fig. 1E). All colour patches have some UV reflectance and tend to increase in reflectance towards longer wavelength (600–700 nm). Beyond 700 nm, both stomatopods and background types tend to have a plateau in reflectance.

**Figure 4:**
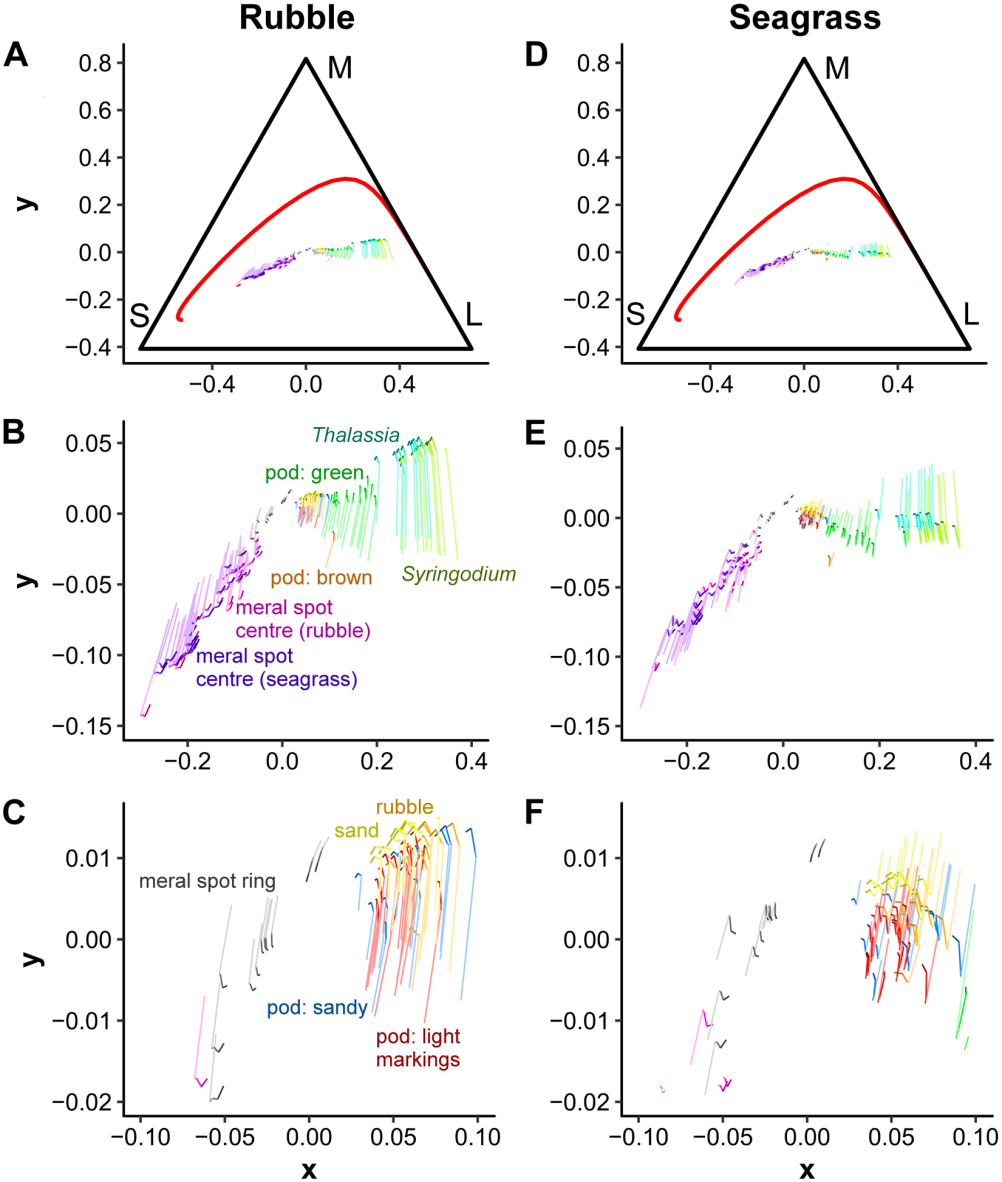
Stomatopod camouflage: hue and saturation. Maxwell’s triangle for a typical fish predator, *Rhinecanthus aculeatus*, viewing habitat and stomatopod (pod) colours modelled using rubble (**A**–**C**) and a seagrass (**D**–**F**) adapting background light. Distance from the centre of the triangle corresponds to saturation and other deviations relate to hue. Red line in (**A**,**D**) is the monochromatic locus and S, M, L indicate the short, medium and long wavelength sensitive photoreceptors, respectively. (**B**,**E**) are zoomed in figures of (**A**,**D**) and (**C**,**F**) are further zoomed in figures of. (**B**,**E**) Each line is a sample, and shading indicates shift in chromatic space with increasing depth from 0.5 m (light) to 10 m (dark).

Across a range of depths, we modelled how stomatopod and habitat colours affect photoreceptor responses of a typical trichromatic fish predator (*R. aculeatus*). Stomatopods and habitat colours are plotted in colour space for the fish predator, called Maxwell’s triangle. Deviations from the centre of the triangle correspond to variation in saturation, and position around the central axis corresponds to hue. All samples tended to fall in a horizontal line across the middle of the Maxwell triangle (Fig. 4). This suggests little variation in relative MWS photoreceptor stimulation across samples. Meral spot centres and meral rings fell towards the SWS photoreceptor in colour space, indicating these samples have relatively more short wavelength reflectance (Figs. 1B, 4). Conversely, habitat colours and stomatopod body colours tend to have relatively more long wavelength reflectance, thus are plotted closer to the LWS photoreceptor (Figs. 1C–F, 4). Sandy stomatopods, light markings on patterned stomatopods, rubble pieces and sand occupy a similar region of colour space, implying a match in hue and saturation for a trichromatic fish predator (Fig. 4B,C). Green stomatopods, however, occupied a region closer to the centre of colour space than both seagrass species, suggesting green stomatopods are less saturated in colour (Fig. 4B). As expected, changes in depth shifted hue, and saturation was reduced at greater than 3–7 m depth depending on the region in colour space (Fig. 4). The shifts with depth in the x, y colour space range from 0.003 units to 0.09 units with a mean value of 0.02 units. In JNDs, this is a range of 0.12 to 1.85 with a mean JND of 0.68.

**Figure 5:**
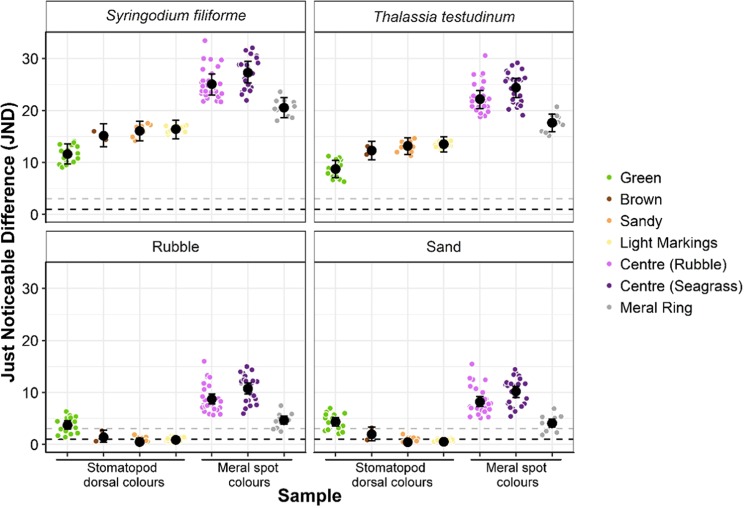
Stomatopod camouflage: Just Noticeable Difference (JND). Contrast between stomatopod colours (dorsal body colours and meral spot colours) and background items (*Syringodium filiforme, Thalassia testudinum*, rubble or sand). Black points and error bars indicate geometric means and 95% bootstrapped confidence intervals for each group. Each coloured point is the mean JND for one sample, compared against each of ten background samples. Dotted black line indicates JND of one (indistinguishable from background) and dotted grey line indicates JND of three (may be indistinguishable).

Spectral contrast calculated from the receptor noise limited model^42^ provides additional insight into predator chromatic discrimination. Following the two-step method described by Maia and White (2018)^45^, samples are likely to be distinct and perceptually discriminable if they are statistically separate and if the confidence intervals of the contrasts do not cross the JND threshold. According to these criteria, all stomatopod colours, including green dorsal, contrasted chromatically with both seagrass species (Table 1, Fig. 5). In rubble and sand habitats, sandy and brown stomatopods are likely chromatically camouflaged because contrasts are below the JND threshold (JND < 3; Fig. 5), and, for brown stomatopods, differences were not statistically separate (Table 1). Meral spot rings and meral spot centres of stomatopods collected from both rubble and seagrass habitats contrasted with sand and rubble backgrounds (Table 1, Fig. 5). It is worth noting that some of the PERMANOVA results may represent a Type I error (Table 1). This occurs when the smaller group has greater variance than the larger group^46^. In particular, the significant result for brown stomatopods in sand habitats likely represents a Type I error because of the low sample size (two brown stomatopods were collected).

**Table 1:** Stomatopod camouflage: hue and saturation.

Sample	Background			
	*Syringodium filiforme*	*Thalassia testudinum*	Rubble	Sand
Green stomatopod	Pseudo-*F*_1,23_ = 121, p = 0.001	Pseudo-*F*_1,23_ = 81.2, p = 0.001	Pseudo-*F*_1,23_ = 36.8, p = 0.001	Pseudo-*F*_1,23_ = 52.8, p = 0.001
Brown stomatopod	Pseudo-*F*_1,10_ = 47.3, p = 0.025	Pseudo-*F*_1,10_ = 43.7, p = 0.025	Pseudo-*F*_1,10_ = 5.70, p = 0.055*	Pseudo-*F*_1,10_ = 8.87, p = 0.031*
Sandy stomatopod	Pseudo-*F*_1,18_ = 241, p = 0.001	Pseudo-*F*_1,18_ = 216, p = 0.001	Pseudo-*F*_1,18_ = 3.40, p = 0.042	Pseudo-*F*_1,18_ = 2.35, p = 0.098
Light markings (patterned stomatopods)	Pseudo-*F*_1,27_ = 549, p = 0.001*	Pseudo-*F*_1,27_ = 515, p = 0.001*	Pseudo-*F*_1,27_ = 12.5, p = 0.002	Pseudo-*F*_1,27_ = 3.38, p = 0.03
Meral spot centre (rubble)	Pseudo-*F*_1,38_ = 580, p = 0.001	Pseudo-*F*_1,38_ = 489, p = 0.001	Pseudo-*F*_1,38_ = 97.6, p = 0.001	Pseudo-*F*_1,38_ = 83.0, p = 0.001
Meral spot centre (seagrass)	Pseudo-*F*_1,32_ = 620, p = 0.001	Pseudo-*F*_1,32_ = 542, p = 0.001	Pseudo-*F*_1,32_ = 156, p = 0.001	Pseudo-*F*_1,32_ = 138, p = 0.001
Meral ring	Pseudo-*F*_1,21_ = 461, p = 0.001^*^	Pseudo-*F*_1,21_ = 435, p = 0.001	Pseudo-*F*_1,21_ = 85.1, p = 0.001	Pseudo-*F*_1,21_ = 59.2, p = 0.001

Results from PERMANOVAs testing significant differences in chromatic contrast between stomatopod colour samples and different backgrounds.

* indicates comparisons where the smaller group has greater variance than the larger group, which can lead to unreliable results and Type I errors (Anderson 2006)^46^.

### Stomatopod camouflage: pattern and total reflectance

We investigated whether green and sandy stomatopods matched the background (seagrass or rubble) in total reflectance, pattern contrast and pattern size, based on objective imaging. As expected, in rubble habitats sandy stomatopods closely match sand and rubble pieces in pattern contrast (*i.e*., pattern energy) and total reflectance (*i.e*., luminance; Cohen’s *d* < 0.8; Table 2). They also match rubble pieces in pattern size (*i.e*., pattern frequency), however they have larger pattern size than sand (Cohen’s *d* = 0.87; Table 2). Sandy stomatopods may also camouflage in seagrass habitats because they have similar pattern contrast, pattern size and total reflectance to sand and *Thalassia* blades covered in silt (Cohen’s *d* < 0.8; Table 2). However, clean *Thalassia* blades were darker and had lower pattern contrast than sandy stomatopods (Cohen’s *d* = 1.61 and 1.56, respectively).

**Table 2:** Stomatopod camouflage: pattern and total reflectance.

	Seagrass Habitat			Rubble Habitat	
	Sand	Clean *Thalassia* blade	*Thalassia* covered in silt	Sand	Rubble pieces
***Sandy stomatopod***					
Pattern contrast	**0.749**	1.556	**0.390**	**0.160**	**0.075**
Pattern size	**0.060**	**0.135**	**0.257**	0.870	**0.453**
Total reflectance	**0.797**	1.605	**0.168**	**0.561**	**0.520**
***Green stomatopod***					
Pattern contrast	1.634	**0.145**	1.174	0.975	1.023
Pattern size	**0.040**	**0.126**	**0.203**	**0.259**	**0.007**
Total reflectance	1.865	**0.148**	1.024	1.476	1.488

Summary of the magnitudes of difference (Cohen’s *d* values) between stomatopod colour variations and different background types, as perceived by a fish predator. Small effect sizes (*d* < 0.8; in bold) indicate ‘matches’ between a stomatopod and background type^58^. These variables were calculated using the green colour channel of the image^53,54^.

Green stomatopods are likely only camouflaged from fish predators in seagrass habitats. Here, they match clean *Thalassia* blades in pattern contrast, pattern size and total reflectance (Cohen’s *d* < 0.8; Table 2). However, green stomatopods are darker and have lower pattern contrast than sand and *Thalassia* blades covered in silt (Cohen’s *d* > 0.8; Table 2). In rubble habitats, they are also darker and have less contrasting patterns than both sand and rubble pieces (Cohen’s *d* > 0.8; Table 2). Surprisingly, green stomatopods have similar pattern size as all background types (Cohen’s *d* < 0.8).

## Discussion

Stomatopods exhibit a huge range of body colours and patterns across species. Despite several studies investigating the signalling role of coloured patches in stomatopod species^20–25,60–62^, we still have little understanding of how stomatopods perceive these signals in their natural environment. Stomatopods are predicted to use a diverse range of visual signals including UV, visible and polarised light. Understanding stomatopod perception of such signals in relation to background colouration and lighting conditions is likely to provide key information about the evolution of stomatopod signals. Here, we demonstrate that variation between habitats in meral spot colour does not increase signal contrast for conspecifics within these habitats. However, contrast between the meral spot center and the meral ring may allow for more accurate assessment of meral spot center total reflectance. In addition, although many stomatopod species also exhibit different dorsal colours and patterns, the potential role of such coloration in providing camouflage has not previously been assessed. By modelling perception of stomatopod colour and pattern by a typical fish predator, we also demonstrate that *N. oerstedii* colour variations likely provide camouflage in seagrass and rubble habitats.

### Stomatopod signalling: meral spot

Previously we discovered that *N. oerstedii* collected from rubble habitats have lighter meral spot centres than those collected from seagrass habitats^25^. Our visual analysis indicates that this variation does not function to increase signal contrast with the background of the local habitat. If this variation did increase contrast, we would expect the contrast between meral spot centres and rubble background to be greater for stomatopods collected from rubble habitats than those from seagrass habitats, and vice versa. However, we observed that stomatopod meral spot centres contrasted more with the habitat that they were not collected from. The variation in total reflectance may be a by-product of altered prey availability in different habitats. Purple colouration in crustaceans is usually based on carotenoid pigments^63,64^, which are pigments that must be ingested^65^. In another stomatopod species, prey abundance has been shown to vary between seagrass and rubble habitats^66^ and this is predicted to influence stomatopod diet. If prey differ in their carotenoid content, it may influence the pigmentation of the meral spot centre. Stomatopods with darker meral spots have been shown to have greater weapon performance^27^ and stomatopods assess meral spot total reflectance during contests^24^. Therefore, carotenoids and diet may link meral spot colour with weapon performance, creating an honest signal. Future studies could investigate whether the relationship between meral spot total reflectance and weapon performance is consistent across habitats.

The meral ring provided greater contrast with seagrass backgrounds than meral spot centres, but contrasted far less with rubble backgrounds than meral spot centres. Contrast is an essential component of signal detectability^1,67^. Thus, the meral ring may improve detectability in seagrass habitats, but may not improve detectability in rubble habitats. Instead, to maximise contrast in rubble habitats stomatopods may have behavioural adaptions similar to those reported in other species. For example, male golden-collared manakins increase contrast between their colourful plumage and the background by clearing away leaf litter from their display arenas^68^. Alternatively, male guppies choose to signal in lighting environments that maximise the contrast of their visual signal^69^. It does not appear that stomatopods increase contrast by modifying the background around the refuge entrance because reflectance measurements are similar between rubble entrances and the top of a piece of rubble (Fig. S4). Contrast may also be enhanced in rubble habitats if meral ring reflectance varies with habitat. We did not have data to assess this possibility in the current study, but variation in reflectance with habitat may be an important mechanism to enhance contrast in each habitat.

Contrast within the meral spot (*i.e*., between meral spot centres and the meral ring) may assist receiver stomatopods to assess the quality of the meral spot. As expected, the meral ring has greater contrast with darker meral spot centres (stomatopods from seagrass habitats) than lighter meral spot centres (stomatopods from rubble habitats). Within pattern colour contrast has been shown to influence receiver behaviour in some organisms^70,71^. For example, female cichlids prefer males with greater pattern contrast^70^. Stomatopods assess the colour of the meral spot during contests over refuges^24,25^ and meral spot total reflectance correlates with stomatopod weapon performance^25^. To determine meral spot centre total reflectance, stomatopods may assess the within pattern contrast, rather than the colour of meral spot centre *per se*. To further investigate within pattern contrast, future studies should record meral spot centre and meral ring reflectance for the same stomatopod to obtain biologically relevant comparisons.

The meral spot is likely an example of a signal that can be hidden from predators, rather than a signal that a predator cannot perceive. Meral spots contrasted with both seagrass and rubble habitats to a fish predator, indicating that stomatopods are not using a secret chromatic signalling channel. Instead, the meral spots are hidden during most stomatopod behaviours because they are located on the inner side of the raptorial appendage. Such signals are common in many taxa and are expected to only be exposed in low predation conditions^9,72,73^. Because this display is visible to fish predators, it is also possible that the meral spots act as a deimatic or startle display against predators^74,75^. Deimatic displays involve conspicuous and rapid changes in behaviour or appearance that are thought to confuse or alarm predators, providing time for prey to escape^75^. Some stomatopods, such as *Gonodactylus smithii*, perform the meral spread to almost any moving object, whereas other species are more cautious (pers. obs.). *Neogonodactylus oerstedii* will perform the meral spread at researchers in the lab, and some have also performed the meral spread at us in the field. However, it is unknown whether they also display in response to predators, and, if so, whether it would be effective as a threat or deimatic display.

### Stomatopod camouflage: pattern and colour

The dorsal body pattern and total reflectance of *N. oerstedii* likely provides camouflage from fish predators. Sandy stomatopods match rubble, sand and *Thalassia* blades covered in silt, and green stomatopods match clean *Thalassia* blades. This suggests that sandy stomatopods may receive camouflage in both seagrass and rubble habitats whereas green stomatopods are likely only camouflaged in seagrass habitats. This is supported by our collection data for this study: we collected a high proportion of sandy stomatopods in both habitats (rubble: 82% collected were sandy; seagrass: 39% collected were sandy) but green stomatopods were only common in seagrass habitats (rubble: 8% collected were green; seagrass: 47% collected were green). The patterns on sandy stomatopods may be a form of disruptive coloration, serving to break up the stomatopod’s outline and make it more difficult for a fish predator to recognise the stomatopod^76,77^. Green stomatopods, however, are likely matching a relatively uniform *Thalassia* seagrass blade. Because seagrass blades are constantly moving with the waves, matching a seagrass blade may provide camouflage whilst stationary or in motion^78,79^. It is predicted that organisms can remain cryptic whilst in motion if the background is moving, they match the background, and they move similarly to the background^78,79^. Green stomatopods may be able to move around in seagrass habitats without detection more freely than sandy stomatopods can move around in rubble habitats.

Within the visual range of a typical trichromatic fish predator, the results of our chromatic contrast analysis suggest that sandy stomatopods match sand and rubble in hue and saturation, whereas green stomatopods do not match either seagrass species in saturation. Therefore, this model predicts that trichromatic fish predators may be able to detect green stomatopods by chromatic differences. However, the spectra of green stomatopods and both seagrass species are extremely similar (Fig. 1C,E), with a peak around 550 nm and plateau above 700 nm. This suggests that the mathematical difference may not translate to an ecologically relevant difference. Furthermore, under the canopy of seagrass, light is dappled and filtered by seagrass blades for photosynthesis^80^. The dappled light may make it difficult for a fish predator to accurately distinguish stomatopod colour and the filtering of light may affect the chromaticity of the green stomatopods. In addition, some organisms use achromatic mechanisms for discrimination when targets are small or far away^81–83^. If fish use achromatic mechanisms when searching for prey, our results suggest green stomatopods would be indistinguishable from *Thalassia* blades since they match in total reflectance.

We also assessed whether changes in incident light at different water depths affects the degree of stomatopod camouflage as perceived by a typical fish predator. Our modelling results predict that such changes affect hue more than saturation. As depth increased, all samples tended to shift in a clockwise direction around the origin when modelled with rubble adapting background light, and anti-clockwise when modelled with seagrass adapting background light. Saturation was only slightly reduced at depths below three to seven meters, depending on the region in colour space. These shifts in hue and saturation had minimal effect on the relative positions in colour space of stomatopod and background colours, *i.e*., green stomatopods remained in the same position relative to both seagrass species across 0.5–10 m depths. Thus, stomatopod background matching remains constant at the range of depths tested. The shifts predicted are similar to those reported for two species of damselfish^16^. Wilkins and colleagues^16^ modelled shifts in colour from 0 m to 30 m depth and note that shifts in colour space vary in direction and magnitude for different colour samples. Our results are slightly more predictable in direction, but magnitude varies substantially among stomatopod and background colours. Our shifts in colour space may be slightly more predictable due to the specific spectral sensitivities of *R. aculeatus* and/or because spectral sensitivities of *R. aculeatus* are more closely spaced than either damselfish species in the other study^16,84^.

## Conclusions

We have shown that stomatopods deal with the opposing pressures of signalling and camouflage by employing a signal that can be hidden from predators and using primary body colouration for camouflage. Stomatopods use a variety of chromatic and achromatic signals during intraspecific interactions^23–25,60–62^. Further investigations into stomatopod signals will likely uncover other signals that can be concealed from predators, as well as secret signals, and signalling behaviours that maximise signal contrast. Furthermore, stomatopods may prove a fruitful system to improve our understanding of fish prey search behaviours. Considerable variation described here in stomatopod body patterns, colours and habitats allow for investigations into various aspects of camouflage, including disruptive camouflage, camouflage whilst moving, and whether fish use achromatic or chromatic vision for prey search.

## Supplementary information

Supplementary figures.
