# Supplementary material for: Differences in signal contrast and camouflage among different colour variations of a stomatopod crustacean, Neogonodactylus oerstedii
Source: Sci Rep. 2020 Jan 27;10:1236. doi: 10.1038/s41598-020-57990-z (PMC6985165; doi:10.1038/s41598-020-57990-z)
Supplement: Supplementary file 1 — Supplementary figures. [file 41598_2020_57990_MOESM1_ESM.docx]

**Differences in signal contrast and camouflage among different colour variations of a stomatopod crustacean, *Neogonodactylus oerstedii***

**Amanda M. Franklin, Justin Marshall, Adina D. Feinstein, Michael J. Bok, Anya D. Byrd, Sara M. Lewis**

**Supplemental figures and results**

**
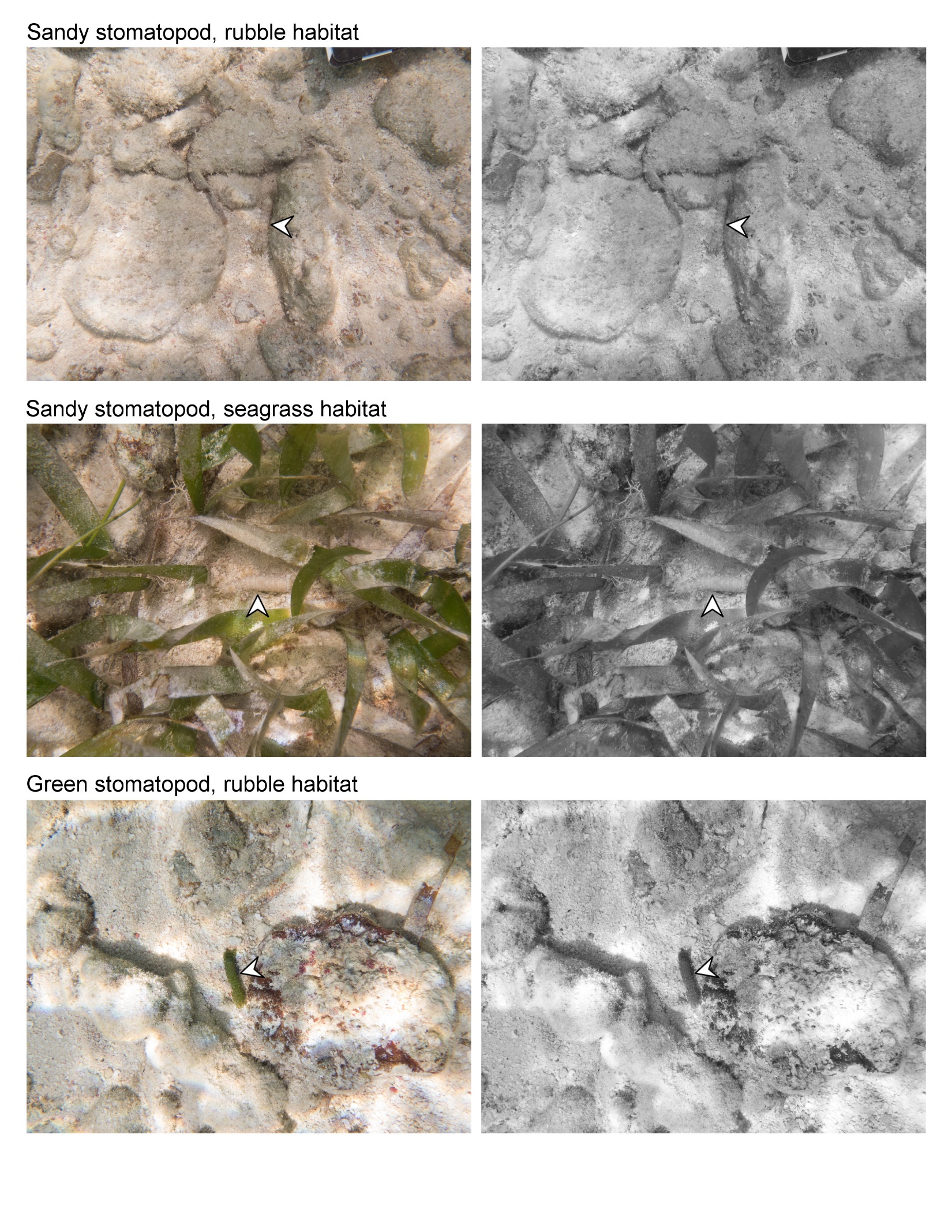
**

**
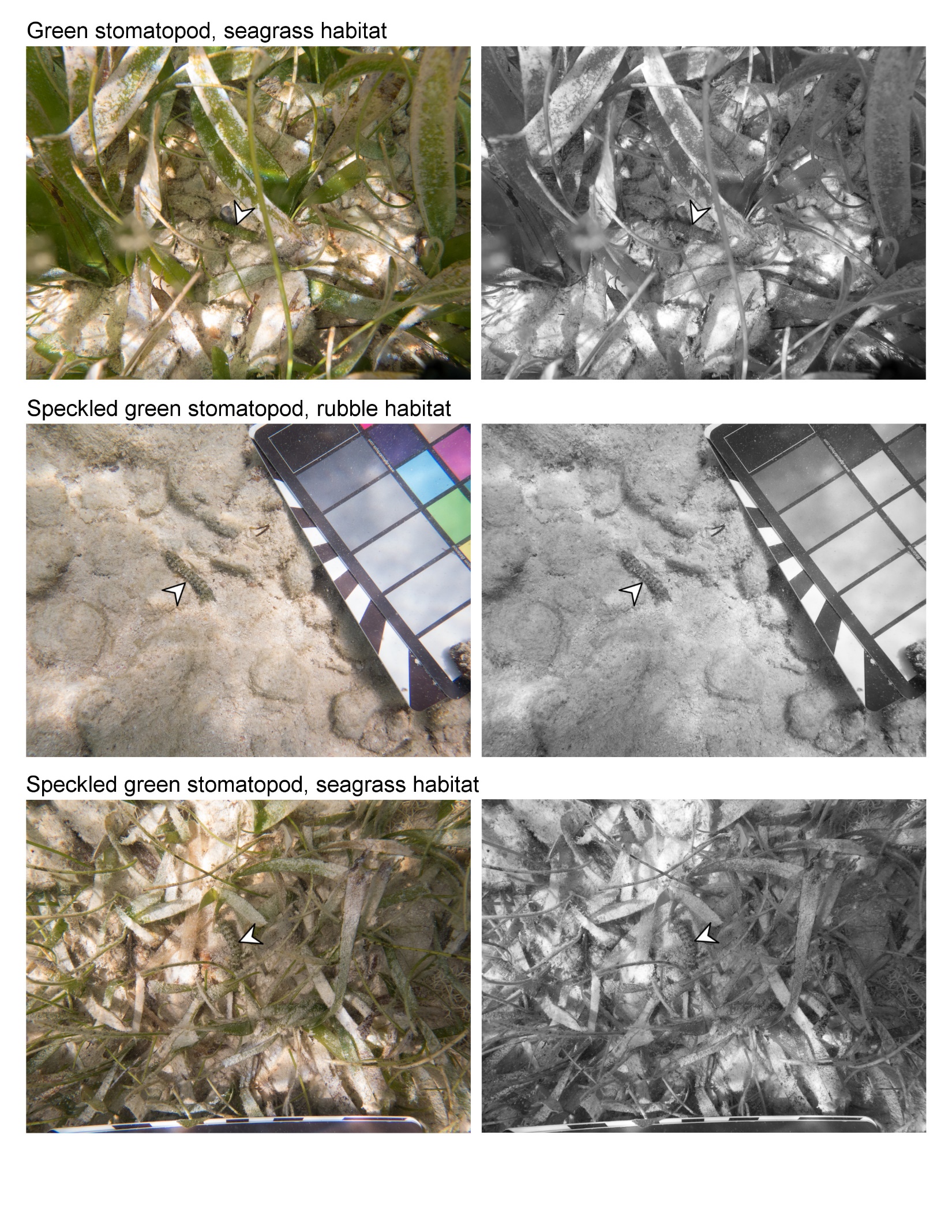
**

**Figure S1: Photographs of sandy, green and speckled green *N. oerstedii* colour morphs in seagrass and rubble habitats.** Colour images are shown on the left and green colour channel images used for pattern and total reflectance analyses are shown on the right. White arrow indicates stomatopod, and is pointing to the left side, midway down the abdomen.


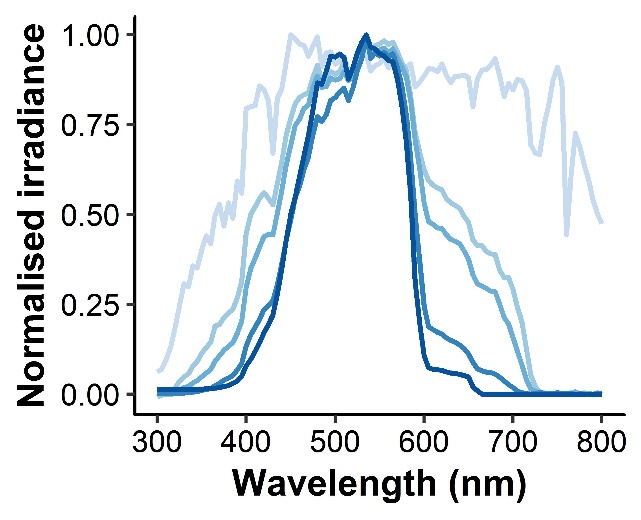


**Figure S2: Irradiance at five depths used for estimates of quantum catch.** Shading from light to dark indicates shallow to deep irradiance (0.5 m, 1 m, 3 m, 7 m, 10 m depth). Irradiance measurements were adapted from Marshall *et al*. (2003).


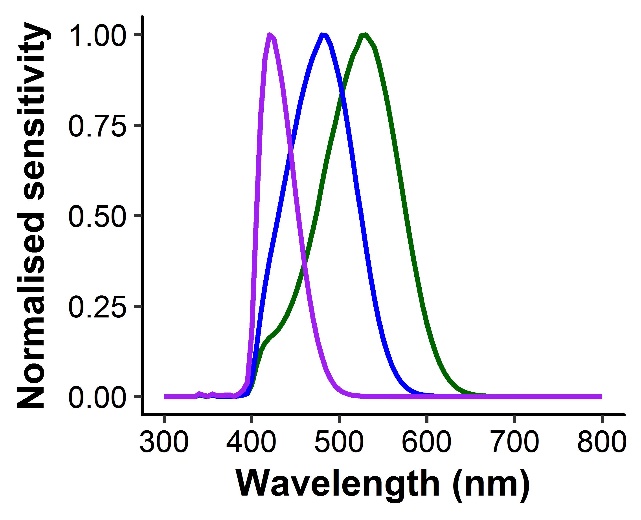


**Figure S3: Spectral sensitivities of the Picasso triggerfish, *Rhinecanthus aculeatus.*** Purple, blue and green lines indicate the short (420 nm), medium (480 nm) and long (520 nm) wavelength sensitive photoreceptors, respectively. Sensitivities were adapted from Pignatelli *et al*. (2010).

1. Top of rubble piece B) Rubble at cavity opening


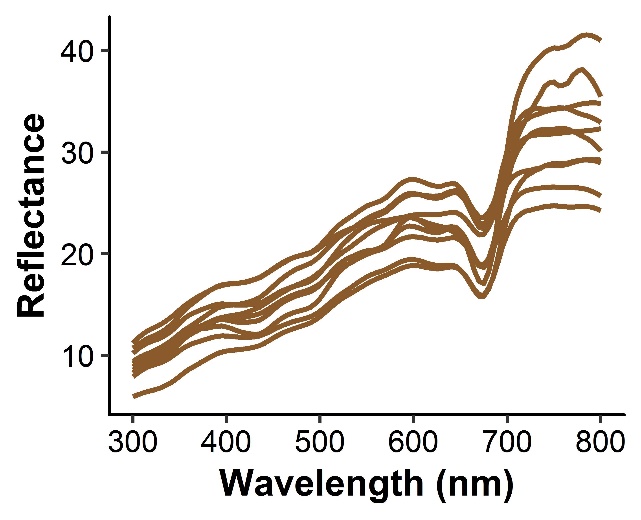

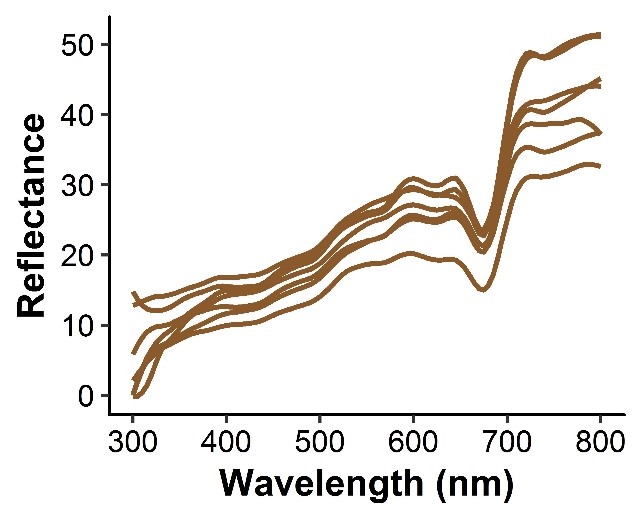


**Figure S4: Spectral reflectance of rubble pieces taken from the top surface (A) or near the opening of stomatopod cavities (B).** Each line is 2- 6 spectra from a single rubble piece that were smoothed and averaged. Spectra of rubble openings were recorded at midday in full sun, whereas spectra of the top of rubble pieces was recorded in the lab with a PX-2 xenon light source (see methods).

**Supplemental references**

Marshall, N., Jennings, K., McFarland, W., Loew, E., Losey, G. & Montgomery, W. (2003) Visual biology of Hawaiian coral reef fishes. III. Environmental light and an integrated approach to the ecology of reef fish vision. *Copeia*, **2003**, 467-480.

Pignatelli, V., Champ, C., Marshall, J. & Vorobyev, M. (2010) Double cones are used for colour discrimination in the reef fish, *Rhinecanthus aculeatus*. *Biology Letters*, rsbl20091010.
